# RAS‐association domain family 1A regulates the abnormal cell proliferation in psoriasis via inhibition of Yes‐associated protein

**DOI:** 10.1111/jcmm.16489

**Published:** 2021-05-07

**Authors:** Jinjing Jia, Ning Wang, Yan Zheng, Xiumei Mo, Yu Zhang, Siqi Ye, Junfeng Liu, Fenggen Yan, Hongyi Li, Dacan Chen

**Affiliations:** ^1^ State Key Laboratory of Dampness Syndrome of Chinese Medicine The Second Affiliated Hospital of Guangzhou University of Chinese Medicine Guangzhou China; ^2^ Department of Dermatology The Second Affiliated Hospital of Guangzhou University of Chinese Medicine Guangzhou China; ^3^ Guangdong Provincial Key Laboratory of Chinese Medicine for Prevention and Treatment of Refractory Chronic Disease Guangzhou China; ^4^ Department of Dermatology The Second Affiliated Hospital of Xi'an Jiaotong University Xi'an China

**Keywords:** abnormal cell proliferation, methylation, psoriasis, RAS‐association domain family, Yes‐associated protein

## Abstract

Psoriasis is a chronic, inflammatory skin disease with a high incidence and recurrence; however, its exact pathogenesis and aetiology remain unclear. This study aimed to analyse the effect of the upstream negative regulator RAS‐association domain family 1A (RASSF1A) on Yes‐associated protein (YAP) in psoriasis. Skin lesions of 22 patients with psoriasis and 19 healthy controls were used. Human epidermal keratinocytes stimulated by M5 (IL‐1α, IL‐17, IL‐22, TNF‐α and oncostatin M) were used to establish a psoriatic cell model. BALB/c mice treated with topical imiquimod were used to establish a psoriatic mouse model. As the methylation level of RASSF1A increased, its expression in psoriatic patients and mice model decreased. Addition of the methylation inhibitor 5‐Aza‐CdR or RASSF1A‐overexpressing lentivirus vector increased RASSF1A and reduced YAP expression; meanwhile improved skin lesions, reduced cell proliferation, induced cell cycle arrest in the G0/G1 phase, increased apoptosis, reduced inflammatory cytokines and activities of ERK, STAT3 and NF‐κB signalling pathways. The results indicated that RASSF1A could play a role in the treatment of psoriasis by inhibiting YAP expression. Based on these findings, targeted drugs that can inhibit the methylation or increase the expression of RASSF1A may be useful for treating psoriasis.

## 1 INTRODUCTION

Psoriasis is a chronic, inflammatory skin disease with a population incidence of 0.1%‐4% and affects all ages and genders equally.^1^, ^2^ Because of its high incidence, recurrence and treatment resistance, psoriasis is considered as a persistent skin disease affecting the physical and mental health of the patients.^3^, ^4^ Currently, the exact pathogenesis and aetiology of psoriasis are unclear. Abnormal proliferation of keratinocytes (KCs) and shortening of cell cycles are its important pathophysiological characteristics. Studies have shown that KCs in psoriasis are apoptosis resistant; therefore, it is important to explore the causes of imbalance in KC apoptosis and cell cycle acceleration in the pathogenesis of psoriasis.^5^ In our previous study, we found that Yes‐associated protein (YAP) is highly expressed in psoriatic lesions and is involved in the pathogenesis of psoriasis through the regulation of KC proliferation and apoptosis.^6^ RAS‐association domain family 1A (RASSF1A), the most common negative regulator of RAS, is expressed in most normal tissues but is down‐regulated or absent in some cell lines and tissues of tumours.^7^

The core of the kinase cascade of the Hippo signalling pathway includes Mst1/2, Sav1, Lats1/2, Mob1, YAP and TAZ. The signalling transduction process of Hippo pathway is composed of a series of kinase cascade phosphorylation reactions and YAP is its downstream effector,^8^ which regulates cell proliferation and apoptosis, and regulates the growth, development, and size of tissues and organs. When phosphorylated by upstream kinases at serine 127, pS127‐YAP is inactivated and retained in the cytoplasm, preventing it from functioning as a transcription factor.^8^, ^9^ However, the relationship between RASSF1A and the Hippo‐YAP signalling pathway remains unclear. Studies have demonstrated that *RASSF1A* is rarely mutated, and its low or loss of expression is mainly due to hypermethylation of the CpG island in the promoter region.^10^ As a negative upstream regulator of Hippo‐YAP signalling pathway,^11^, ^12^, ^13^ RASSF1A may play an important role in the pathogenesis of psoriasis. Several research groups have showed that in mammalian cells, RASSF1A can interact with Mst, one of the core of the kinase cascade in the Hippo‐YAP signalling pathway. Furthermore, RASSF1A can interact with Mst1/2 through the SARAH domain,^11^, ^12^ but the role of RASSF1A in combination with Mst1/2 was unclear before 2007. Matallanas et al^13^ found that the oncogene *Raf* can bind and inhibit the activity of Mst2, leading to inhibition of the following apoptotic signalling pathway. RASSF1A can inhibit the interaction between *Raf* and Mst2, leading to phosphorylation of Mst2 and further to the phosphorylation of Lats1/2 and downstream YAP. Phosphorylated YAP can bind to p73 and promote the transcription of *PUMA*, which promotes apoptosis. In addition, by activating the Hippo signalling pathway, RASSF1A leads to activation of Fas, TNF‐α or BAX‐mediated apoptosis. Knockdown of RASSF1A leads to a decrease in Mst1/2 activation and blockage of apoptosis. The previous study examined RASSF1A expression and methylation in psoriasis, analysed its correlation with YAP expression and verified its effect on YAP expression, KC proliferation, cell cycle, and apoptosis by modifying the RASSF1A methylation level in vitro and in vivo.

## 2 MATERIALS AND METHODS

### 2.1 Patient samples

Skin lesions of 22 patients with psoriasis (12 males and 10 females) were collected from the Department of Dermatology, the Second Affiliated Hospital of Xi'an Jiaotong University from September 2016 to August 2019, and 19 normal skin tissues (10 males and 9 females) from cosmetic surgeries were selected as controls. The study was approved by the ethics committee of the Second Affiliated Hospital of Xi'an Jiaotong University and informed consents were obtained. All the psoriatic specimens were confirmed by pathological examinations. Additionally, 10 psoriasis samples and 10 healthy control samples from the collected samples were frozen in liquid nitrogen for subsequent detection of protein and methylation levels.

### 2.2 Histology and immunohistochemistry

Tissue samples were paraffin‐embedded and sectioned. Hematoxylin and eosin (HE) and immunohistochemical stainings were performed following a standard staining procedure.^14^ The thickness of the skin lesions was measured using Image Pro‐Plus 6.0 software (Media Cybernetics). Based on the results of the HE staining examined under microscope, the average thickness was determined from mean values in three microscopic fields. The results of immunohistochemistry analysis were classified as negative (−), weakly positive (+), moderately positive (++) and strongly positive (+++) after double‐blind examination by two pathologists.

### 2.3 Establishing the psoriatic cell model

Human epidermal KCs (ScienCell) were routinely cultured in KC medium (ScienCell). Recently, KCs or KC cell lines stimulated with the M5 mixture (mixture of IL‐1 α, IL‐17, IL‐22, TNF‐α and oncostatin M) were recognized as the psoriatic cell models.^15^ In the present study, we used 10 ng/mL M5 (PeproTech) to stimulate KCs for 48 hours. The stimulated KCs were divided into three groups, which were then treated with different concentrations of 5‐Aza‐CdR (5, 10, 20 μmol/L; MedChemExpress), a widely used methylation inhibitor.

### 2.4 Lentivirus infection

An RASSF1A overexpression vector (the CMV promoter is shown in Figure S1A) was constructed by Shenyang Wanleibio Technology. Cells in the logarithmic growth period were infected with lentivirus (multiplicity of infection [MOI] = 200) according to the manufacturer's instructions. The expression of RASSF1A and YAP was detected 48 hours after infection.

### 2.5 Establishing the psoriatic mouse model

Currently, using topical imiquimod on the backs of mice for seven consecutive days is universally acknowledged as the method to establish a psoriatic animal model.^16^ Twenty‐five 6‐ to 8‐week‐old female BALB/c mice (18‐20 g) were routinely raised in specific pathogen‐free conditions. All animal experiments were approved by the ethics committee of the Second Affiliated Hospital of Xi'an Jiaotong University. The mice were randomly divided into five groups: control, psoriasis and three 5‐Aza‐CdR groups (treated with a different concentration of 5‐Aza‐CdR). In the psoriasis groups, 62.5 mg of imiquimod cream (Hubei Keyi Pharmaceutical Co., Ltd.) was applied daily to the back of the mice. In the control group, an equal amount of Vaseline (Nanchang Baiyun Pharmaceutical Co., Ltd.) was applied daily. In the 5‐Aza‐CdR group, different concentrations (5, 10 and 20 μmol/L) of 5‐Aza‐CdR were applied besides imiquimod application. At the end of 7 days, mice were anaesthetized with phenobarbital (Shenyang Wanleibio Co., Ltd.) and euthanized.

### 2.6 Quantitative real‐time PCR (qRT‐PCR)

TRIzol (Invitrogen) was used to extract total RNA from cells or skin tissues, and cDNA was obtained by reverse transcription. The sequences of the primers synthesized by Shanghai Biotechnology Co., Ltd. are shown in Supplemental Table S1. The standard curves for each group were obtained through qRT‐PCR. After standardization to glyceraldehyde‐3‐phosphate dehydrogenase levels, relative YAP expression was calculated using the 2^−ΔΔ^ *^CT^* method. The experiment was repeated three times, and three technical replicates were included for each data point.

### 2.7 Western blotting

Total protein from cells or tissues was extracted and quantified. Twenty‐five micrograms of proteins was separated by 10% polyacrylamide gel electrophoresis, and then transferred onto a nitrocellulose membrane. Skim milk (5%) was used to block non‐specific antigen binding, and then primary antibodies were added (RASSF1A, ab23950, Abcam; YAP, #14074, Cell Signaling Technology; pS127‐YAP, #13008; Cell Signaling Technology; Cyclin A, sc‐596, Santa Cruz Biotechnology; Cyclin B1, 55004‐1‐AP, Proteintech; Cyclin D1, sc‐246, Santa Cruz Biotechnology; Cyclin E, sc‐25303, Santa Cruz Biotechnology; CDK1, WL02373, Wanleibio; cleaved‐caspase‐3, WL02348, Wanleibio; Bcl‐2, WL01556, Wanleibio; BAX, 50599‐2‐lg, Proteintech; p73, WL01604, Wanleibio; p53, sc‐126, Santa Cruz Biotechnology; p21, WL0362, Wanleibio; AKT, WL0003b, Wanleibio; p‐AKT, WLP001a, Wanleibio; ERK, WL01864, Wanleibio; p‐ERK, WLP1512, Wanleibio; STAT3, WL03207, Wanleibio; p‐STAT3, WLP2412, Wanleibio; NF‐κB P65, WL01980, Wanleibio; LATS1/2, YT2543, ImmunoWay Biotechnology; p‐LATS1/2, YP1222, ImmunoWay Biotechnology; MST1/2, 37462, Signalway Antibody; p‐MST1/2, bs‐3294R, Bioss; β‐actin, sc‐47778, Santa Cruz Biotechnology; Histone H3, Wanleibio) and incubated at 4°C overnight. Next, secondary antibodies were added, followed by incubation at room temperature for 1 hour and subsequent exposure to an X‐ray film.

### 2.8 Detection of RASSF1A gene promoter methylation

Tissue DNA was extracted according to the manufacturer's instructions (Bioteke). Methylation of the RASSF1A promoter was detected through methylation‐specific PCR (MSP). DNA samples were treated using an EZ DNA methylation kit (ZYMO Research). Then, PCR assay and agarose gel analysis were performed. Primer sequences of methylated RASSF1A (M‐RASSF1A) and unmethylated RASSF1A (U‐RASSF1A) are shown in Table S2.

### 2.9 Cell proliferation assay

Cells were inoculated into a 96‐well plate at the concentration of 5 × 10^3^ cells/200 μL culture medium per well. MTT (20 μL) was added to each well 24, 48 and 72 hours later. After 4 hours of incubation in the dark, 150 μL of dimethyl sulphoxide was added to each well, and the plate was shaken for 10 minutes. The absorbance value of each well at 570 nm was measured. Five technical replicates were evaluated for each group. Experiments were repeated three times, and five technical replicates were performed for each data point.

### 2.10 Cell cycle

Cell cycle was measured according to the manufacturer's instructions (Jiangsu Kaiji Biotechnology Co., Ltd.). The cells were digested and fixed overnight with 75% ethanol at −20°C. Next, 100 μg/mL RNaseA and 50 μg/mL propidium iodide (PI) staining solution were added, and the cells were incubated at 37°C in the dark for 30 minutes. The cell cycle was analysed by flow cytometry (FACSCalibur, BD Biosciences). Three independent experiments were performed.

### 2.11 Cell apoptosis

Cell apoptosis was measured according to the manufacturer's instructions (Jiangsu Kaiji Biotechnology Co., Ltd.). Approximately, 1‒5 × 10^5^ cells were suspended in 500 μL binding buffer, stained with 5 μL Annexin V‐FITC and 5 μL PI staining solution, and incubated in the dark for 5‒15 minutes at room temperature. The cell apoptosis was measured by flow cytometry. Three independent experiments were performed.

### 2.12 ELISA

The concentrations of IL‐1 β, IL‐6, IL‐17 and IL‐23 from supernatant of culture medium or mouse serum samples were determined through standard ELISA using an ELISA kit according to the manufacturer's instructions (Lianke Biotech). Optical density (OD) values at 450 nm were then obtained using a spectrophotometer to construct the standard curve. Experiments were repeated three times, and three technical replicates were included for each data point.

### 2.13 Statistical analysis

All data are presented as the mean ± standard error of the mean. SPSS 19.0 software (SPSS, Inc.) was used for statistical analysis. Pearson chi‐square and Mann‐Whitney *U* tests were used to analyse the immunohistochemistry results. Unpaired Student's *t* test was used for comparisons between two groups. One‐way analysis of variance was used for experiments performed with 3 or more groups and the *LSD* test was used as a post hoc for multiple comparisons. *P* < .05 was considered statistically significant.

## 3 RESULTS

### 3.1 Expression and methylation of RASSF1A in psoriasis

**FIGURE 1:**
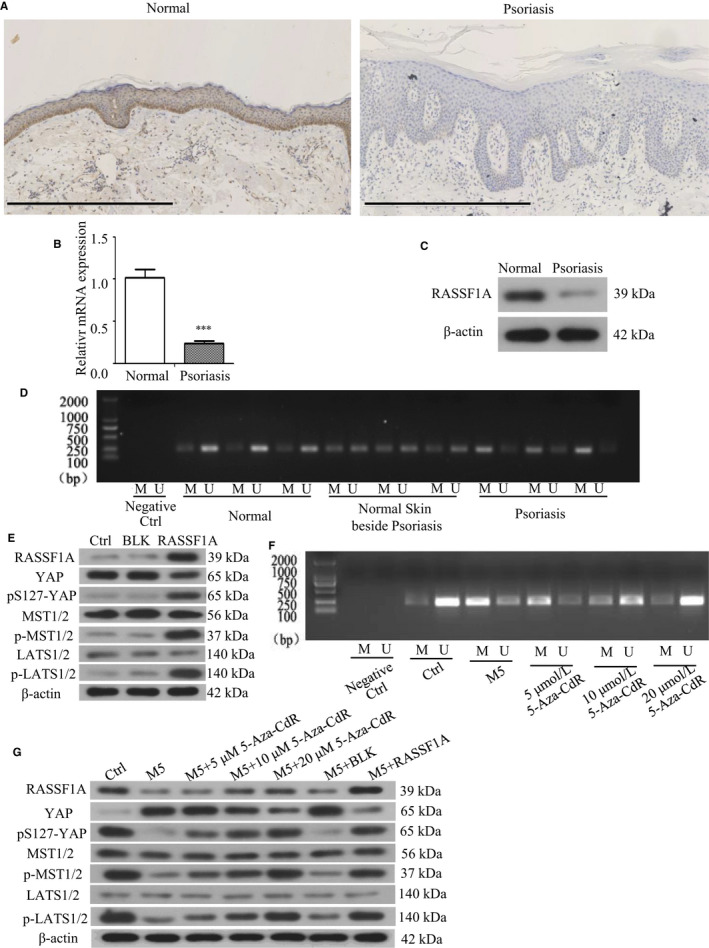
Expression and methylation of RASSF1A in the skin lesions and effect of RASSF1A‐overexpressing lentivirus infection and methylation inhibitor 5‐Aza‐CdR. A, Immunohistochemistry of RASSF1A in psoriasis and normal skin tissues (bar length = 600 μm). B, RASSF1A mRNA expression in psoriasis and normal skin tissues (n = 3). C, RASSF1A protein expression in psoriasis and normal skin tissues. D, RASSF1A methylation in psoriasis and normal skin tissues. E, RASSF1A, YAP, pS127‐YAP, LATS1/2, p‐LATS1/2, MST1/2 and p‐MST1/2 protein expression after RASSF1A overexpression lentivirus transfection. F, RASSF1A methylation after adding methylation inhibitor 5‐Aza‐CdR. G, RASSF1A, YAP, pS127‐YAP, LATS1/2, p‐LATS1/2, MST1/2 and p‐MST1/2 protein expression in M5‐induced psoriatic cells after adding 5‐Aza‐CdR or RASSF1A overexpression lentivirus. M = methylated RASSF1A, U = unmethylated RASSF1A. BLK = empty lentivirus‐transfected group. RASSF1A = RASSF1A overexpression lentivirus‐transfected group. ***P < .001 vs Normal

The immunohistochemistry results showed that the expression rate of RASSF1A protein was 78.95% (15/19) in normal skin tissues and 50.00% (11/22) in psoriatic lesions (Table 1, Figure 1A). The qRT‐PCR and Western blot results showed that RASSF1A mRNA and protein expression in normal skin were higher than that in psoriatic lesions (Figure 1B,C). MSP assays showed that the methylation level of RASSF1A increased gradually in normal skin tissue, normal tissue beside psoriatic lesions and psoriatic tissues, indicating that the low expression of RASSF1A may be due to promoter methylation (Figure 1D).

**TABLE 1:** RASSF1A expression in psoriasis and normal skin tissues

Group	N	Expression Grade				Positive rate (%)
		−	+	++	+++	
Normal	19	4	5	4	6	78.947
Psoriasis	22	11	6	4	1	50.000*

* *P* < .05 compared with normal skin.

### 3.2 Validation of RASSF1A overexpression efficiency and its effect on YAP expression

To confirm the effect of RASSF1A overexpression on YAP expression, we infected cells with a RASSF1A‐overexpressing lentivirus. After 48 hours of lentivirus infection, RASSF1A mRNA and protein were overexpressed in the group infected with the RASSF1A overexpression lentivirus compared to their expression in the control group (Ctrl) and the group infected with empty virus vector (BLK). RASSF1A overexpression reduced YAP mRNA and protein expression and induced pS127‐YAP, p‐LATS1/2 and p‐MST1/2 expression (Figure 1E; Figure S1B,C).

### 3.3 Effect of 5‐Aza‐CdR on RASSF1A and YAP expression in psoriatic cells

The MSP results showed that different concentrations of 5‐Aza‐CdR dose‐dependently reduced the RASSF1A methylation level in psoriatic cell model induced by M5 (Figure 1F). Western blotting showed that 5‐Aza‐CdR dose‐dependently increased RASSF1A expression in psoriatic cells; the level did not return to pre‐M5 levels. Simultaneously, 5‐Aza‐CdR gradually reduced YAP expression and induced pS127‐YAP, p‐LATS1/2 and p‐MST1/2 expression in M5‐induced psoriatic cells. RASSF1A overexpression in psoriatic cells had similar effects as 5‐Aza‐CdR (Figure 1G).

### 3.4 Effect of RASSF1A overexpression and 5‐Aza‐CdR on cell proliferation

**FIGURE 2:**
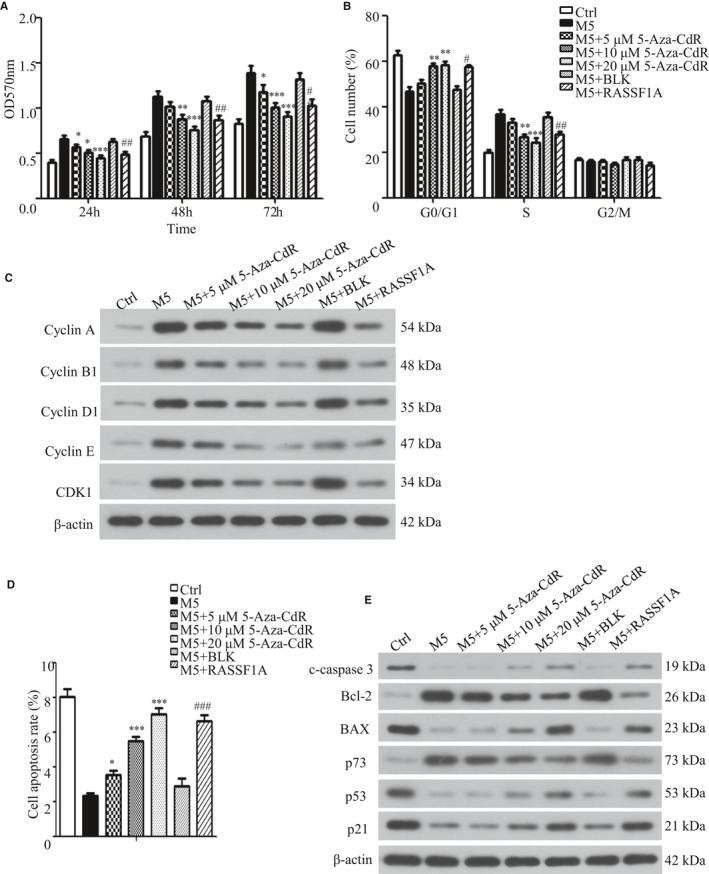
Effect of overexpression of RASSF1A and 5‐Aza‐CdR on cell proliferation, cell cycle and apoptosis. A, Cell proliferation rate 24, 48 and 72 h after adding methylation inhibitor 5‐Aza‐CdR or transfecting RASSF1A overexpression lentivirus (n = 3). B, Cell cycle profiles were analysed at 48 h after adding 5‐Aza‐CdR or transfecting RASSF1A overexpression lentivirus (n = 3). C, Cell cycle regulators were analysed by Western blotting at 48 h after adding 5‐Aza‐CdR or transfecting RASSF1A overexpression lentivirus. D, Cell apoptosis profiles were analysed at 48 h after adding 5‐Aza‐CdR or transfecting RASSF1A‐overexpressing lentivirus (n = 3). E, Apoptosis‐related proteins were analysed by Western blotting at 48 h after adding 5‐Aza‐CdR or transfecting RASSF1A‐overexpressing lentivirus. BLK = empty lentivirus‐transfected group. RASSF1A = RASSF1A overexpression lentivirus‐transfected group. *P < .05, **P < .01, ***P < .001 vs M5; ##*P* < .01, ###*P* < .001 vs M5 + BLK

The results of MTT showed that cell proliferation increased at all time points after adding M5, which is consistent with the characteristic of abnormal proliferation in psoriasis. 5‐Aza‐CdR dose‐dependently inhibited cell proliferation, but the level did not return to pre‐M5 levels. The cell proliferation ability also increased following incubation with M5. After RASSF1A overexpression, the proliferation of cells decreased significantly, but did not return to pre‐M5 levels (Figure 2A).

### 3.5 Effect of RASSF1A overexpression and 5‐Aza‐CdR on cell cycle

Results showed that the proportion of cells in the G0/G1 phase decreased and that of cells in the S phase increased after adding M5, indicating that the proportion of actively replicating cells was increased. This is consistent with the abnormal psoriasis cell proliferation model (Figure 2A). Adding 5‐Aza‐CdR increased the G0/G1 phase ratio and decreased the S phase ratio, which became more obvious with increasing 5‐Aza‐CdR concentrations but did not return to pre‐M5 levels. There was no significant difference in the cell cycle distribution between the BLK‐transfected group and non‐transfected group. However, after RASSF1A overexpression, the proportion of cells in the G0/G1 phase increased and that of cells in the S phase decreased but did not return to pre‐M5 levels. Although the G2/M ratio also changed, there was no significant difference (Figure 2B; Figure S1D). Western blotting showed that the expression of cyclin A, cyclin B1, cyclin D1, cyclin E and CDK1 increased to different degrees when M5 was added. Their expression decreased in a dose‐dependent manner when 5‐Aza‐CdR was added; RASSF1A overexpression had the same effect (Figure 2C). Cell cycle regulation is complex because different regulatory factors are involved in each cell cycle. These results showed that altering RASSF1A expression affected the cell cycle of psoriatic cells by affecting the expression of each cell cycle regulation factors.

### 3.6 Effect of RASSF1A overexpression and 5‐Aza‐CdR on cell apoptosis

The results showed that the KC apoptosis rate decreased when M5 was added and increased when 5‐Aza‐CdR or RASSF1A was added, but did not return to pre‐M5 levels (Figure 2D; Figure S1E). Further, Western blot analysis showed that expression of the apoptosis‐related proteins cleaved‐caspase‐3 (c‐caspase‐3), BAX, p53 and p21 was decreased, whereas that of Bcl‐2 and p73 was increased. RASSF1A overexpression or 5‐Aza‐CdR partially restored this expression to different degrees (Figure 2E).

### 3.7 Effect of methylation inhibitor 5‐Aza‐CdR on psoriatic mouse model

**FIGURE 3:**
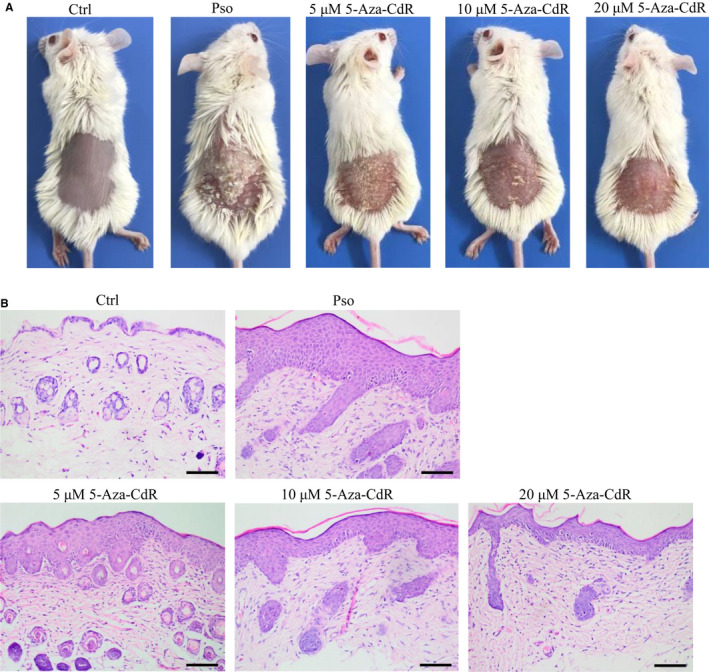
Effect of methylation inhibitor 5‐Aza‐CdR on psoriatic mouse model. A, General images of mice. B, HE staining of histopathological features (bar length = 100 μm)

Our in vitro results confirmed that RASSF1A overexpression by lentivirus infection or treatment with 5‐Aza‐CdR inhibited cell proliferation and induced G0/G1 cell cycle arrest and apoptosis in the M5‐induced psoriatic cell model. To verify the results in vivo, we established an imiquimod‐induced psoriatic mouse model. The skin of the psoriatic model group (Pso) mice was thickened with erythema and scales, whereas the skin of Ctrl mice was smooth, indicating that model induction was successful. The erythema and scales on the mouse's back decreased, and the skin lesions became thinner with increasing 5‐Aza‐CdR concentrations (Table 2, Figure 3A,B).

**TABLE 2:** Thicknesses of the skin tissues of mice

Group	N	Skin thickness (µm)
Ctrl	5	23.21 ± 2.66
Pso	5	169.76 ± 21.36
5 μmol/L 5‐Aza‐CdR	5	156.65 ± 11.58
10 μmol/L 5‐Aza‐CdR	5	129.68 ± 12.08*
20 μmol/L 5‐Aza‐CdR	5	71.10 ± 7.07**

* *P* < .05.

** *P* < .001 vs Pso.

### 3.8 Effect of 5‐Aza‐CdR on YAP and RASSF1A expression in psoriatic mouse model

**FIGURE 4:**
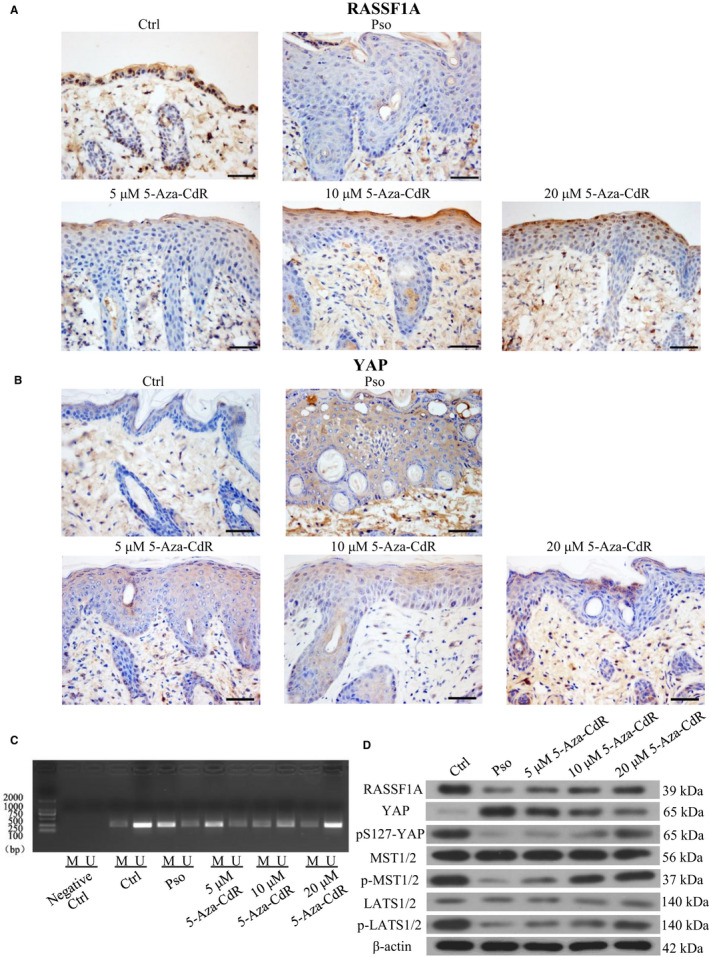
Effect of methylation inhibitor 5‐Aza‐CdR on methylation or expression of RASSF1A and YAP in psoriatic mouse model. A, Immunohistochemistry of RASSF1A expression (bar length = 50 μm). B, Immunohistochemistry of YAP expression (bar length = 50 μm). C, RASSF1A methylation. M = methylated RASSF1A, U = unmethylated RASSF1A. D, RASSF1A, YAP, pS127‐YAP, LATS1/2, p‐LATS1/2, MST1/2 and p‐MST1/2 protein expression in the skin tissues of mice

Immunohistochemistry analysis confirmed that RASSF1A expression in the Pso was lower than that in the Ctrl in mice (Figure 4A) and similar to that in human samples (Figure 1A). Addition of 5‐Aza‐CdR increased the positive rate of RASSF1A expression, and the trend was more obvious with increasing 5‐Aza‐CdR concentrations. Semiquantitative analysis showed that the intensity of RASSF1A expression in the epidermis of the Ctrl group significantly differed from that in the Pso group (*P* = .032). Moreover, the expression rate of YAP in the Pso group was higher than that in the Ctrl group. The positive rate of YAP expression decreased after adding 5‐Aza‐CdR, and the trend was more obvious with increasing 5‐Aza‐CdR concentrations (Figure 4B). Semiquantitative analysis of the expression intensity showed that RASSF1A expression in the Ctrl group was significantly higher than that in the Pso group (*P* = .009), and there was a significant difference between the Pso group and the 10 μmol/L 5‐Aza‐CdR group (*P* = .031) or 20 μnol/L 5‐Aza‐CdR group (*P* = .013) (Table 3). MSP confirmed that 5‐Aza‐CdR reduced RASSF1A methylation (Figure 4C), and Western blotting confirmed that 5‐Aza‐CdR induced RASSF1A expression while reducing YAP expression and driving pS127‐YAP, p‐LATS1/2 and p‐MST1/2 in a dose‐dependent manner (Figure 4D).

**TABLE 3:** Results of immunohistochemistry of RASSF1A and YAP in mouse skin lesions

Group		N	Expression grade				Positive rate (%)
			−	+	++	+++	
RASSF1A	Ctrl	5	1	0	1	3	80
	Pso	5	4	1	0	0	20
	5 μmol/L 5‐Aza‐CdR	5	3	2	0	0	40
	10 μmol/L 5‐Aza‐CdR	5	2	3	0	0	60
	20 μmol/L 5‐Aza‐CdR	5	2	2	1	0	60
YAP	Ctrl	5	4	1	0	0	20
	Pso	5	0	1	1	3	100*
	5 μmol/L 5‐Aza‐CdR	5	1	2	1	1	80
	10 μmol/L 5‐Aza‐CdR	5	2	2	1	0	60
	20 μmol/L 5‐Aza‐CdR	5	3	2	0	0	40**

* *P* <.01 vs Ctrl.

** *P* <.05 vs Pso.

### 3.9 Effects of RASSF1A overexpression and 5‐Aza‐CdR on cytokine expression

**FIGURE 5:**
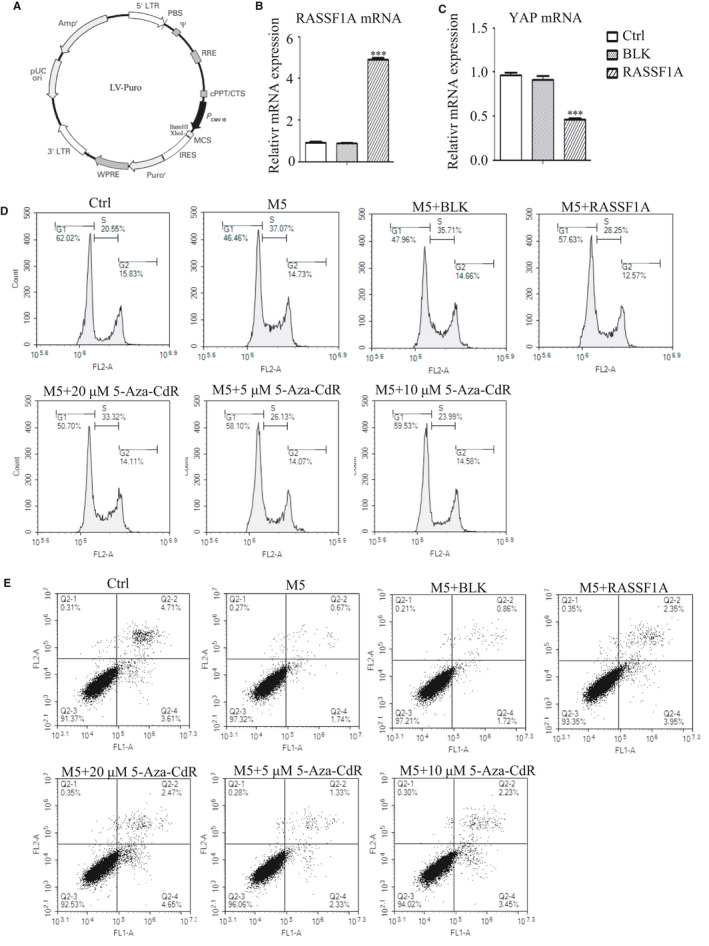
Effect of overexpression of RASSF1A and 5‐Aza‐CdR on the expression of cytokines or signalling pathways. A, IL‐1β, IL‐6, IL‐17 and IL‐23 in the cell culture supernatant 48 h after adding methylation inhibitor 5‐Aza‐CdR or transfecting RASSF1A‐overexpressing lentivirus in M5‐induced psoriatic cell model (n = 3). B, IL‐1β, IL‐6, IL‐17 and IL‐23 in mouse serum (n = 3). C, Protein expression of key members of the AKT, ERK, STAT3 and NF‐κB signalling pathways. BLK = empty lentivirus‐transfected group. RASSF1A = RASSF1A overexpression lentivirus‐transfected group. *P < .05, **P < .01, ***P < .001 vs M5; ###*P* < .001 vs M5 + BLK

ELISA results showed that the concentrations of IL‐1β, IL‐6, IL‐17 and IL‐23 in the supernatant of the psoriatic model of cells or mouse serum were increased. RASSF1A overexpression or addition of 5‐Aza‐CdR to the cells and topical 5‐Aza‐CdR administration on the back of mice reduced cytokine expression to different degrees but the levels did not return to the Ctrl level (Figure 5A,B). This suggests that RASSF1A reduces the expression of these cytokines at different degrees to reduce the inflammatory response in psoriasis.

### 3.10 Effect of RASSF1A overexpression and 5‐Aza‐CdR on the expression of various signalling pathways

To explore which signalling pathways are involved in the effect of RASSF1A on psoriasis, expression of the key members of the AKT, ERK, STAT3 and NF‐κB pathways was analysed through Western blotting. Results showed that the expression of p‐ERK, p‐STAT3 and NF‐κB P65 in the nucleus (NF‐κB P65[n]) was increased and that of NF‐κB P65 in the cytoplasm (NF‐κB P65[p]) was decreased upon addition of M5 to KC cells under conditions where the total AKT, ERK and STAT3 levels were basically unchanged, indicating increased activities of these signalling pathways. However, RASSF1A overexpression or addition of the methylation inhibitor 5‐Aza‐CdR reduced the expression of p‐ERK, p‐STAT3, NF‐κB P65 (n) and increased that of NF‐κB P65 (p); changes in the expression of p‐AKT were less obvious (Figure 5C).

## 4 DISCUSSION

Our previous study showed that YAP expression increased in psoriasis patients and psoriasis skin lesions of mouse models. In vitro, after YAP knockdown, the proliferation of the KC cell line HaCaT was slowed, the cell cycle was blocked in G0/G1 phase, and cell apoptosis was increased.^6^ However, it remains unknown which upstream regulator controls YAP expression in psoriasis.

In this study, we found that the expression of RASSF1A decreased, whereas its methylation increased in psoriasis lesions compared to that in normal skin tissues. Thus, we predicted that a relationship exists between the expression of RASSF1A and YAP. We assumed that loss of RASSF1A expression is mainly caused by hypermethylation of the CpG island in the promoter region^10^; therefore, we used the method of inhibiting RASSF1A methylation or RASSF1A‐overexpressing lentivirus infection to overexpressed RASSF1A expression. After RASSF1A was overexpressed by lentivirus infection or treatment with 5‐Aza‐CdR, YAP expression was found to be down‐regulated, along with up‐regulation of pS127‐YAP, p‐LATS1/2 and p‐MST1/2 protein expression, indicating that RASSF1A controls YAP expression through phosphorylation of the Hippo signalling pathway. It is known that YAP is active in its non‐phosphorylated form, which can enter the nucleus and initiate a series of gene transcriptions. After phosphorylation, YAP is inactivated and retained in the cytoplasm, and thus, it cannot play the role of a transcription factor.^17^, ^18^ The down‐regulation of YAP and up‐regulation of pS127‐YAP indicate the inactivation of YAP. Simultaneously, the proliferation rate of M5‐induced psoriatic cells decreased; the cell cycle was blocked in the G0/G1 phase and the expression of the cell cycle regulators (cyclin A, cyclin B1, cyclin D1, cyclin E and CDK1) was decreased. Further, under the same conditions, apoptosis was increased and the expression of the apoptosis‐promoting proteins cleaved‐caspase‐3, BAX, p53 and p21 was increased, whereas the expression of the apoptosis‐inhibiting protein Bcl‐2 was decreased. Changes in the psoriatic cell model after RASSF1A overexpression were consistent with those observed after YAP knockdown.^6^ Analysis of related signalling pathways showed that after RASSF1A overexpression, the expression of key molecules in the STAT3, ERK and NF‐κB signalling pathways was down‐regulated, with less obvious changes in AKT signalling activity. In vivo evaluation of imiquimod‐induced psoriatic mice confirmed that 5‐Aza‐CdR improved skin lesions, increased RASSF1A expression and reduced YAP expression. It may be possible that YAP is expressed at a higher level in the cytoplasm than in the nucleus in KCs, which is why the cytoplasmic expression of YAP was more obvious in immunohistochemistry results. Further, the levels of IL‐1β, IL‐6, IL‐17 and IL‐23 in the supernatant of the M5‐induced psoriatic cells or mouse serum were decreased, indicating suppression of the inflammatory response. Our previous studies showed that YAP promoted cell proliferation, invasion and migration,^19^ although the upstream regulator remained unclear. Psoriasis shares some features with tumours, such as immortalized abnormal proliferation. As RASSF1A overexpression inhibited YAP expression in both cell and animal experiments, RASSF1A may inhibit psoriatic cell proliferation, induce apoptosis and reduced the expression of inflammatory factors by inhibiting YAP expression via the Hippo signalling pathway. Increasing RASSF1A expression using methylation inhibitors may provide new strategies for developing drug therapies for psoriasis. However, the specific regulatory process of the RASSF1A‐Hippo‐YAP signalling pathway requires further analysis.

## CONFLICT OF INTEREST

The authors confirm that there are no conflicts of interest.

## AUTHOR CONTRIBUTION

**Jinjing Jia:** Conceptualization (equal); Funding acquisition (equal); Investigation (equal); Writing‐original draft (equal). **Ning Wang:** Investigation (equal); Methodology (equal). **Yan Zheng:** Conceptualization (equal); Investigation (equal). **Xiumei Mo:** Validation (equal); Writing‐review & editing (equal). **Yu Zhang:** Investigation (equal); Methodology (equal). **Siqi Ye:** Investigation (equal); Methodology (equal). **Junfeng Liu:** Data curation (equal); Formal analysis (equal). **Fenggen Yan:** Data curation (equal); Formal analysis (equal). **Hongyi Li:** Validation (equal); Writing‐review & editing (equal). **Dacan Chen:** Supervision (equal); Validation (equal).

## Supporting information

Supplementary Material

## Data Availability

No data have been shared during the current study.
