# Supplementary material for: RAS‐association domain family 1A regulates the abnormal cell proliferation in psoriasis via inhibition of Yes‐associated protein
Source: J Cell Mol Med. 2021 May 7;25(11):5070–81. doi: 10.1111/jcmm.16489 (PMC8178269; doi:10.1111/jcmm.16489)
Supplement: Supplementary file 1 — Supplementary Material [file JCMM-25-5070-s001.doc]

**Supplementary materials**

**Supplemental Table S1.** The qRT-PCR primers used in this study

| Gene name | Sequences |
| --- | --- |
| YAP | Forward: 5ʹ- GCTAGACCCAAGGCTTGACC-3ʹ  Reverse: 5ʹ- ATTTGCTGTGCTGGGATTGA-3ʹ |
| RASSF1A | Forward: 5ʹ- GTTCACCTGCCACTACCGC-3ʹ  Reverse: 5ʹ- AAGGTCAGGTGTCTCCCACT-3ʹ |
| GAPDH | Forward: 5ʹ- CACTGTGCCCATCTACGAGG-3ʹ |
|  | Reverse: 5ʹ- TAATGTCACGCACGATTTCC-3ʹ |

**Supplemental Table S2.** The MSP primers used in this study

| Gene name | Sequences |
| --- | --- |
| M-RASSF1A | Forward: 5ʹ-TAAGTGTGTTGTTTTAGTAAATCGG-3ʹ  Reverse: 5ʹ-AAAAACTATAAAACCCGAAAACGA-3ʹ |
| U-RASSF1A | Forward: 5ʹ-TAAGTGTGTTGTTTTAGTAAATTGG-3ʹ |
|  | Reverse: 5ʹ-AAAACTATAAAACCCAAAAACAAA-3ʹ |


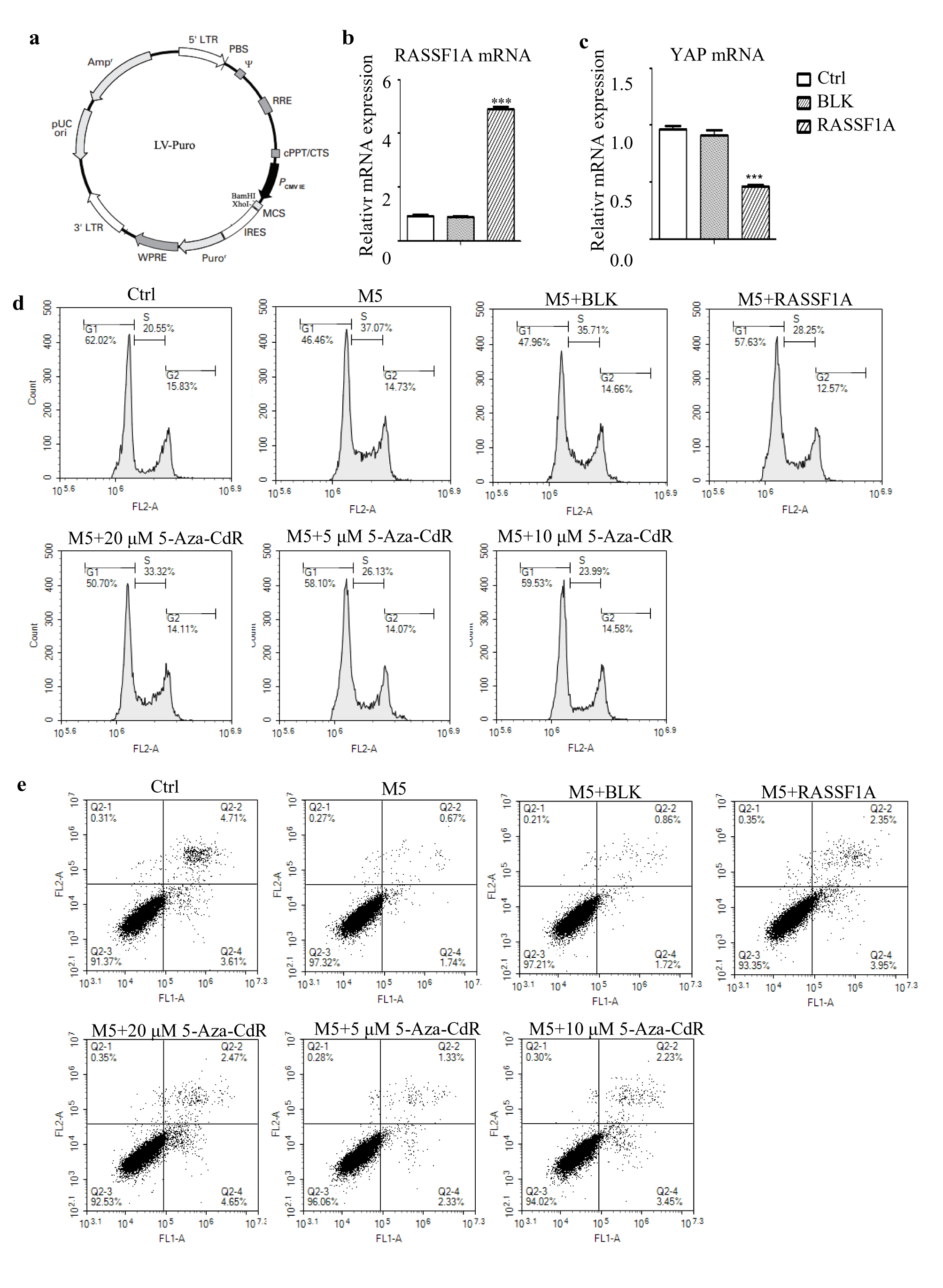


**Supplemental Fig. S1. a** Information of the RASSF1A overexpression vector. **b** RASSF1A mRNA expression after RASSF1A overexpression lentivirus transfection. **c** YAP mRNA expression after RASSF1A overexpression lentivirus transfection. **d** The cell cycle profiles were analyzed 48 h after adding methylation inhibitor 5-Aza-CdR or transfecting RASSF1A overexpression lentivirus. **e** The cell apoptosis profiles were analyzed 48 h after adding methylation inhibitor 5-Aza-CdR or transfecting RASSF1A overexpression lentivirus.
